# Anal HPV/HIV co-infection among Men Who Have Sex with Men: a cross-sectional survey from three cities in China

**DOI:** 10.1038/srep21368

**Published:** 2016-02-19

**Authors:** Xiangwei Li, Mufei Li, Yu Yang, Xiang Zhong, Boxuan Feng, Henan Xin, Zhen Li, Qi Jin, Lei Gao

**Affiliations:** 1 MOH Key Laboratory of Systems Biology of Pathogens, Institute of Pathogen Biology, Chinese Academy of Medical Sciences & Peking Union Medical College, Beijing, China; 2 Department of Epidemiology and Biostatistics, School of Public Health, Peking University Health Science Centre, Beijing, China; 3 MSD China Holding Co., Ltd, Beijing, China

## Abstract

Anal human papillomavirus (HPV) infection, which is linked to anal warts and anal cancer, is common among men who have sex with men (MSM), especially among those HIV positives. MSM aged 18 years or older were recruited from mainland China. Blood and anal cytologic samples were collected for HIV-1 serological test and HPV genotyping. A total of 889 eligible participations were included. The genotyping results of HPV were available for 822 participants (92.46%), 65.32% of which were positive for the targeted 37 HPV types. Prevalence of infection with any type of HPV was higher among HIV-infected participants (82.69%) as compared to HIV-uninfected participants (62.81%) (p < 0.01). HPV06 (15.45%), HPV18(13.50%), HPV16 (11.44%), and HPV11 (10.71%) were the most frequent types identified in the study population. Ever had paid sex with man was found to be independent predictor for HPV positivity with an adjusted odds ratios (OR) of 2.34 (1.16–4.74). Anal HPV positivity was observed to be independently associated with HIV infection with an adjusted OR of 3.03 (1.76–5.21) in the study population. In conclusion, the prevalence of anal HPV infection was observed to be common among MSM from mainland China, and it was significantly associated with the status of HIV infection.

Anal infection with human papillomavirus (HPV) is extremely common in men who have sex with men (MSM)^1^,^2^,^3^. Although most HPV infections are transient and clinically unapparent, close attention has been paid to HPV because of its causal link to certain cancers. Besides the established association between high-risk HPV infection and cervical cancer, persistent HPV infection has also been suggested to be an important risk factor for the development of several other cancers, such as laryngeal cancer, esophageal cancer and anal cancer^4^,^5^,^6^. Our previous study showed that 62.10% of the MSMs from Beijing and Tianjin cities had been infected with HPV, including several oncogenic HPV types such as 16, 18, 31, 33, 35, 45, 52, and 58^7^. In addition, previous studies show that nearly all of the HIV-positive MSM were infected with HPV, which was approximately 1.5 times higher than HIV-negative MSM^7^,^8^. A recent systematic review and meta-analysis which included 21 studies of HIV-positive MSM reported a summary prevalence estimate of HPV infection any type to be 92.6% (90.8–94.5)^9^. Epidemiologic assessment indicated that behavioral factors might play roles in the prevalence, incidence, and persistence of anal HPV infection, such as the mounts of sex partners, unprotected anal sex, and cigarette smoking^7^,^8^,^10^.

In recent years, the prevalence of HIV/AIDS and other sexually transmitted diseases (STDs) in China have been rapidly rising among MSM, which draws great concern of the public health fields^11^,^12^. Stigma and discrimination related to homosexual activities and HIV/STD infection have been the major barriers to MSM seeking health services^13^. In China, homosexuality is still regarded as deviation from mainstream society despite of an increasing acceptance of MSM in general society. Stigma and social pressure for MSM in China were reported to mainly arise from their families to get married and have children to protect family reputation and lineage. This might increase the risk of HIV spread from the high-risk population to general population. The study by Zhang Lei and colleagues indicated HIV prevalence in MSM in China increased from 1.77% (1.26–2.57) in 2000, to 5.98% (4.43–8.18) in 2010, with a national incidence of 0.98 (0.70–1.25) per 100 person-years in 2010^14^. Therefore, the prevalence of anal HPV infection and its association with HIV among this high-risk population should be more widely addressed in China. It is important to examine how empirical HPV findings in Chinese MSM relate to other international studies. Based on our previous studies conducted in Beijing and Tianjin, the objectives of this cross-sectional study were investigating the status of anal HPV infection and HIV infection among MSM from other cities in China and identifying the most prevalent HPV genotypes.

## Results

### Subject recruitment and characteristics

In total, 889 eligible participations were interviewed and signed the informed consents, 289 from Chengdu, 300 from Xi’an, and 300 from Taiyuan, respectively. Blood sample and anal swab were successfully collected from each of them. As shown in Table 1, major characteristics of the study participants were evaluated by site. Nearly half of them were younger than 30 years (47.25%), 70.53% reported homosexual tendency, 10.02% reported a history of genital wart, and 11.02% reported other STDs history other than genital wart and HIV. Laboratory data suggested a prevalence of 12.26% of HIV seropositivity among the study population.

### Anal HPV type distribution

As shown in Supplementary Table S1 and Table 2, the genotyping results of HPV were available for 822 participants (92.46% of all), 65.32% of them were positive for at least one of the targeted 37 HPV types. Prevalence of infection with any type of HPV was higher among HIV-infected participants (82.69%), as compared to HIV-uninfected participants (62.81%) (p < 0.01).Among HPV positives, 41.53% (223/537) of them were infected with multiple types. Among HIV-positived participants, 17.31% had none HPV subtypes detected, 34.62% were positive for only one type, and 48.08% were detected with two or more types. HPV06 (15.45%), HPV18 (13.50%), HPV16 (11.44%), and HPV11 (10.71%) were found to be the most frequently identified types. The prevalence of HPV types of 56, 82, 52, 39, 68, 51, 31, 66, 45, 33, 55, 83, 6, 61, 40 and 44 were found to be significantly higher among HIV-infected compared to uninfected participants. In addition, the distribution pattern of the genotypes was found to be various among the study sites (Supplementary Table S1).

### Risk factors for anal HPV infection

The associations of anal HPV infection with potential risk factors were assessed by logistic regression analysis as shown in Table 3. Covariates with *P* < 0.1 in univariate models were adjusted for in a multiple logistic regression model. Ever had paid sex with man in the past year was found to be significantly related to HPV seropositivity with an adjusted OR of 2.34 (1.16–4.74).

### Risk factors for HIV infection

As shown in Table 4, condom use during insertive anal sex in the past 6 months, ever found sexual partners in gay venues and anal HPV infection were significantly associated with HIV seropositivity in univariate analyses. The variables were included multiple logistic regression analysis with *P* < 0.1 in univariate models. Anal HPV statuses were significantly associated with HIV seropositivity with an adjusted OR of 3.03 (1.76–5.21).

### The association between HIV seropositivity and anal HPV infection

As shown in Table 5, we analyzed the association between HIV status and anal HPV infection according to HPV oncogenic type and the number of co-infected HPV types. There are 73 and 346 subjects were infected with at least one High-risk subtypes among the HIV-positive and HIV-negative groups, respectively. The results showed both low-risk and high-risk HPV infection were found to be significantly related to HIV seropositivity. There was a significant association between HPV multiplicity and the prevalence of HIV.

## Discussion

The present study demonstrated a high prevalence of anal HPV infection among MSM from three cities of China. The prevalence of anal HPV infection was much common among HIV-infected MSM (82.69%) than HIV negatives (62.81%). HPV18 and 16 were most frequently identified high-risk types and HPV06 and 11 were most frequently identified low-risk types, respectively. The prevalence of HPV genotypes was found might to be varied between the study sites. Additionally, the prevalence of anal HPV infection was found to be related to the status of HIV infection, which is consistent with our findings and the evidence from other regions^7^,^8^,^15^,^16^,^17^. The definition of High-risk HPV types was mainly according to the manufacturer’s instructions. Meanwhile, we defined the debatable High-risk HPV types by the previous studies^18^,^19^.

Although the prevalence of anal HPV infection among HIV-positive MSM from mainland China was 82.69%, it was slightly lower than those previous studies reported from HIV-positive MSM in the Canada (97.90%)^20^, and was significant higher than a Dutch gay-cohort study reported 65.00%^21^. Moreover, our result of the anal HPV infection prevalence among HIV-positive MSM was similar as a study from Thailand (85%)^17^. This discrepancy might be partly due to mode of life, variation in viral clearance and infection incubation time. For HIV-negative MSM, previous studies from the United States and Latin America showed the overall anal HPV prevalence was ranging from 42% to 66%^2^,^3^,^22^,^23^, which were comparable with our 62.81% anal HPV prevalence.

HPV06 (15.45%), HPV18 (13.50%), HPV16 (11.44%) and HPV11 (10.71%) were found to be the most frequently identified types among study population. But a different type distribution was observed by HIV status, the prevalence of several HPV types was significantly higher among HIV positives than among negatives. It is considered that immunosuppression from HIV infection not only increases risk of acquisition of new HPV infections, but also HPV infection persistence and reactivation of latent infections. The HPV types of 18 and 16 were the most common high-risk HPV types identified in both HIV-positive and HIV-negative MSM in our study. These data were consistent with our previous studies^7^. However, as compared to the study from Thailand^17^, the HPV types of 16 was the most common high-risk HPV types in both HIV-positive and HIV-negative MSM and the HPV18 was less common than HPV06. Our results suggest that the most frequently distributed HPV types among Chinese MSM could be covered by the current commercial HPV vaccines, which provides flexibility for HPV vaccination among this high risk population to prevent HPV infection and related diseases. Different from prior study^24^, in our analysis, former smokers or current smoking was not associated with a statistically significantly elevated risk of any HPV infection. Possibly due to the immunomodulatory properties only could be observed among individuals who are actively and seriously exposed to cigarette smoke.

In association analysis, we found the risk of anal HPV infection was not related to condom use during insertive anal sex in the past 6 months. There are mixed data on whether condoms decrease the incidence of HPV infection. To some extent, this might also influenced by the reason that we did not distinguish condom use during insertive anal sex or receptive anal sex. Several other studies did not find a relation between them either, perhaps because not all the condoms could effectively cover all the exposed skin susceptible to infection^25^,^26^.

In our present study, the subjects HPV infection status were grouped into four subgroups: negative for any type, positive only for high-risk, positive only for low-risk HPV, and positive for both. Our results indicated that low-risk and high-risk HPV were associated with increased HIV seropositivity, and the association for the high-risk HPV was higher. Previous study also demonstrates that anal HPV infection in MSM is associated with HIV acquisition^24^,^27^,^28^. Moreover a recent systemic review and meta-analysis included 6567 participants suggests that HPV-infected individuals have twice the risk of acquiring HIV^29^. However, the mechanisms are not yet clear. Several hypotheses may explain the reason HPV-infected individuals are more susceptibility of to HIV, including lesions infected HPV may be more friable and susceptible to disruption of mucosal, which may promote HIV entry and replication^30^,^31^. Further studies are warranted to clarify the underlying mechanisms for the different HPV type distribution between HIV negatives and positives.

Our study has several limitations. First, the cross-sectional nature of the study precluded us from determining the causal direction of significant associations between anal HPV infection and HIV seropositivity. Second, over reported or underreported sexual behaviors could not be completely excluded in the study, which may have introduced some misclassification of exposure bias into our estimates. Third, study results may have limited generalizability because participants were only recruited from3 cities, and all of the cities were well-developed metropolitan in China. Fourth, it should be kept in mind that HPV infected cells might not be collected successfully using the current sampling method, and accuracy of the test results might also be influenced by the quality of the samples. Fifth, in our study, not all the potential risk or protected sexual behavior factors were collected. For example, we did not distinguish condom use during insertive anal sex or receptive anal sex. Nonetheless, our study raises serious concern for HPV infection in MSM in China and demonstrates the importance to control HPV/HIV co-infection.

In conclusion, high prevalence of anal HPV infection was observed among MSM from mainland China. Anal HPV infection was identified to be significantly associated with HIV seropositivity. Moreover, with respect to the high prevalence of HPV/HIV co-infection in MSM, studies on anal and cervical HPV diseases in women should be done to gradually extend our observations seen here among men. With the US FDA approval of the HPV prophylactic vaccine, immunization in HPV-unexposed individuals may have the potential to reduce the risk of HPV and HIV acquisition as well. Therefore, quicken up introduction of vaccines to China market might reduce the incidence HPV and HIV to some extent. And above all, improving behavior intervention and health care in the high risk population should be paid more attention in China.

## Materials and Methods

### Ethic statement

The study was approved by the Ethics Committees of the Institute of Pathogen Biology, Chinese Academy of Medical Sciences & Peking Union Medical College. Written informed consent was obtained from each study participant before the interview and testing. All methods were carried out according to the approved guidelines set by The Institute of Pathogen Biology, Chinese Academy of Medical Sciences & Peking Union Medical College.

### Study site and participants

We performed a cross-sectional study of HPV prevalence using anal swab specimens among HIV-infected and uninfected MSM. Study participants were recruited from 3 cities (Xi’an, Chengdu, and Taiyuan) between March 2012 and December 2013, through local nongovernment organizations (Xi’an Tongkang Volunteers Workstation, Chengdu Yongle Volunteers Workstation, and Taiyuan kangtong Volunteers Workstation). Multiple methods were used for recruitment including website advertisement, the community, STD clinics, and voluntary HIV counseling and testing (VCT) clinics. Eligibility criteria were: (1) male and at least 18 years old, (2) self-reported ever had sex with men in the nearly past years, (3) willingness to provide blood samples for HIV and anal swab specimens to test HPV infection and genotypes and (4) able and willing to provide written informed consent.

### Data collection

Data were collected using a questionnaire administered by trained interviewers in a private room. Each study participant was assigned a unique code that was used to link the questionnaire. Data were collected, including self-reported sociodemographic characteristics (e.g., age, income, ethnicity, education, employment, and marriage status, smoking habits), sexual behaviors in the past 6 months, knowledge on HPV and HPV vaccine, and the acceptance of the HPV vaccine. Clinical physical examination was performed to detect any genital and anal ulcers and warts. The nature of ulcers and discharges suggestive of sexually transmitted infections (STIs) were described in detail by trained physicians blinded to questionnaire data.

### Sample collection and laboratory tests

The sample collection and physical examination were performed by trained and experienced physicians. Blood samples were collected for test of HIV serology. The HIV infection status was determined by an enzyme immunoassay (Wantai Biological Medicine Company, Beijing, China). And positive tests were confirmed by HIV-1/2 Western blot assay (HIV Blot 2.2 WB; Gene labs Diagnostics, Singapore). Trained health workers at each site collected anal samples by rotating saline water moistened nylon flocked swab in the anal canal for about 2 minutes. The swab was then kept in 3 mL of sample transport medium for Hybribio 37 HPV GenoArray Diagnostic Kit (Chaozhou Hybribio Limited Corporation, Guangzhou, China). Hybribio 37 HPV GenoArray Diagnostic Kit Test is based on a flow-through hybridization and gene-chip method. The low-density gene chip was pre-fixed with 37 type-specific oligonucleotides and the genotype was analyzed using HybriMax (Chaozhou Hybribio Limited Corporation). The results were then evaluated by a colorimetric change on the chip under direct visualization. Blue–purple spots were recognized as HPV positive. This testing kit can detect 37 common types of HPV, including 23 oncogenic (16, 18, 26, 31, 33, 35,39, 45, 51, 52, 56, 58, 59, 66, 68, 82,83,53, 55, 34, 57, 69, and 71) and 14 non-oncogenic types (06, 11, 40, 42, 44, 54,61, 67, 70, 72, 73, 82, and 84)^18^,^19^. Study participants with genotyping positive for any HPV type were determined to be currently infected with HPV.

### Statistical analysis

Data for questionnaire responses, physical examinations, and laboratory tests were entered independently by two study staff and verified with EpiData software (EpiData 3.1 for Windows, The EpiData Association Odense, Denmark). After cleaning, the data were then converted and analyzed using Statistical Analysis System (SAS 9.12 for Windows; SAS Institute Inc., NC, USA).

Descriptive analyses were performed to compare socio-demographic characteristics, and sexual and drug use behaviors between HIV-infected and uninfected participants. Pearson’s chi-square test and Student’s t-test were used to compare differences between HIV-infected and uninfected participants for categorical and continuous variables, respectively.

Unconditional logistic regression models were used to evaluate the association between each variable and anal HPV infection. Multivariable logistic regression was employed to determine predictors of anal HPV infection. All variables with *p*-values < 0.1 in univariate analyses were entered into the multivariate logistic model. Crude odds ratios (OR) and adjusted odds ratios (adjusted OR) were calculated, as appropriate along with 95% confidence intervals (CI).

## Additional Information

**How to cite this article**: Li, X. *et al.* Anal HPV/HIV co-infection among Men Who Have Sex with Men: a cross-sectional survey from three cities in China. *Sci. Rep.* **6**, 21368; doi: 10.1038/srep21368 (2016).

## Supplementary Material

Supplementary Information

## Figures and Tables

**Table 1:** Descriptive Statistics for demographics and behaviors.

Variable		By site			
	Total n (%)	Chengdu	Xi’an	Taiyuan	*P-*value
Total	889	289	300	300	
Age	32.17 ± 9.73	35.32 ± 12.07	31.63 ± 8.05	31.29 ± 8.12	<0.01
≤29 years	420(47.25)	114(39.45)	153(51.00)	153(51.00)	
30–39 years	287(32.28)	99(34.26)	96(32.00)	92(30.67)	
≥40 years	182(20.47)	76(26.30)	51(17.00)	55(18.33)	
Education					
≤9 years	111(12.49)	57(19.72)	37(12.33)	17(5.67)	<0.01
10–12 years	188(21.15)	81(28.03)	49(16.33)	58(19.33)	
>12 years	590(66.37)	151(52.25)	214(71.33)	225(75.00)	
Marriage status					
Unmarried	599(67.38)	194(67.13)	208(69.33)	197(65.67)	0.06
Married	217(24.41)	61(21.11)	74(24.67)	82(27.33)	
Divorced or widowed	73(8.21)	34(11.76)	18(6.00)	21(7.00)	
Self-reported sexual orientation					
Homosexual	627(70.53)	196(67.82)	184(61.33)	247(82.33)	<0.01
Heterosexual	9(1.01)	3(1.04)	4(1.33)	2(0.67)	
Bisexual	219(24.63)	86(29.76)	87(29.00)	46(15.33)	
Not sure	34(3.82)	4(1.38)	25(8.33)	5(1.67)	
Ever had sex with women					
Yes	430(48.37)	161(55.71)	148(49.33)	121(40.33)	<0.01
No	459(51.63)	128(44.29)	152(50.67)	179(59.67)	
Number of lifetime homosexual partners					
1–5	235(26.43)	84(29.07)	89(29.67)	62(20.67)	0.03
6–10	259(29.13)	93(32.18)	83(27.67)	83(27.67)	
11–30	263(29.58)	78(26.99)	81(27.00)	104(34.67)	
More than 30	132(14.85)	34(11.76)	47(15.67)	51(17.00)	
Age at first homosexual behavior (years, mean ± SD)	22.37 ± 6.43	23.30 ± 8.11	22.55 ± 5.65	21.26 ± 5.22	<0.01
Have current sexual partner	376(42.29)	131(45.33)	98(32.67)	147(49.00)	<0.01
Ever been diagnosed with genital wart	90(10.02)	21(7.27)	37(12.33)	32(10.67)	0.12
Ever been diagnosed with other STIs than genital wart	98(11.02)	19(6.57)	56(18.67)	23(7.67)	<0.01
HIV-1 serostatus					
Negative	780(87.74)	247(85.47)	257(85.67)	276(92.00)	0.02
Positive	109(12.26)	42(14.53)	43(14.33)	24(8.00)	

Abbreviation: SD, standard deviation; STIs, sexually transmitted infections.

**Table 2:** Anal HPV type distribution by HIV-1 serostatus.

HPV type	Total (N = 822)	HIV-1 serostatus		
		Negatives (N = 718)	Positives (N = 104)	*P-*value
Any type	537(65.32)	451(62.81)	86(82.69)	<0.01
Any High-risk type	419 (50.97)	346 (48.19)	73 (70.19)	<0.01
Single type	314(38.19)	278(38.72)	36(34.62)	0.42
Multiple types	223(27.12)	173(23.96)	50(48.08)	<0.01
High Risk				
HPV 18	111(13.50)	94(13.09)	17(16.35)	0.36
HPV 16	94(11.44)	81(11.28)	13(12.50)	0.71
HPV 59	78(9.49)	64(8.91)	14(13.46)	0.14
HPV 56	76(9.25)	59(8.22)	17(16.35)	<0.01
HPV 82	42(5.11)	32(4.46)	10(9.62)	0.02
HPV 52	37(4.50)	27(3.76)	10(9.62)	<0.01
HPV 39	34(4.14)	25(3.48)	9(8.65)	0.01
HPV 68	31(3.77)	23(3.20)	8(7.69)	0.02
HPV 58	30(3.65)	25(3.48)	5(4.81)	0.50
HPV 51	29(3.53)	20(2.79)	9(8.65)	<0.01
HPV 31	22(2.68)	14(1.95)	8(7.69)	<0.01
HPV 66	21(2.55)	15(2.09)	6(5.77)	0.02
HPV 45	17(2.07)	8(1.11)	9(8.65)	<0.01
HPV 33	15(1.82)	12(1.67)	3(2.88)	<0.01
HPV 55	13(1.58)	6(0.84)	7(6.73)	<0.01
HPV 53	6(0.73)	5(0.70)	1(0.96)	0.77
HPV 35	5(0.61)	3(0.42)	2(1.92)	0.07
HPV 83	4(0.49)	2(0.28)	2(1.92)	0.02
HPV 26	1(0.12)	1(0.14)	0	/
HPV 57	1(0.12)	1(0.14)	0	/
HPV 34	0	0	0	/
HPV 69	0	0	0	/
HPV 71	0	0	0	/
Low Risk				
HPV 6	127(15.45)	100(13.93)	27(25.96)	<0.01
HPV 11	88(10.71)	74(10.31)	14(13.46)	0.33
HPV 61	26(3.16)	19(2.65)	7(6.73)	0.02
HPV 40	19(2.31)	13(1.81)	6(5.77)	0.01
HPV 44	11(1.34)	4(0.56)	7(6.73)	<0.01
HPV 42	3(0.36)	2(0.28)	1(0.96)	0.28
HPV 84	6(0.73)	6(0.84)	0	/
HPV 81	3(0.36)	3(0.42)	0	/
HPV 54	2(0.24)	2(0.28)	0	/
HPV 43	0	0	0	/
HPV 67	0	0	0	/
HPV 70	0	0	0	/
HPV 72	0	0	0	/
HPV 73	0	0	0	/

**Table 3:** Risk factors associated with anal infection for 37 HPV subtypes.

Variables	Prevalence n/N* (%)	OR (95% CI)	*P-*value	Adjusted OR (95% CI)	*P-*value
Site			0.00		
Chengdu	193/281 (68.69)	Ref.	Ref.	Ref.	Ref.
Xi’an	135/256 (52.73)	0.79 (0.56–1.10)	0.17	0.73 (0.49–1.07)	0.11
Taiyuan	208/285 (72.98)	2.06 (1.48–2.86)	0.00	2.13 (1.48–3.05)	0.00
Age			0.012		
≤29 years	259/392 (66.07)	Ref.	Ref.	Ref.	Ref.
30–39 years	170/261 (65.13)	0.91 (0.64–1.29)	0.59	0.79 (0.50–1.24)	0.30
≥40 years	108/169 (63.91)	1.00 (0.69–1.47)	0.82	0.99 (0.65–1.52)	0.97
Education			0.002		
≤9 years	71/105 (67.62)	Ref.	Ref.	Ref.	Ref.
10–12 years	115/170 (67.65)	0.89 (0.55–1.44)	0.63	0.82 (0.48–1.38)	0.45
>12 years	351/547 (64.17)	1.07 (0.77–1.50)	0.68	1.29 (0.87–1.91)	0.21
Marriage status			0.20		
Unmarried	361/551 (65.52)	Ref.	Ref.		
Married	134/205 (65.36)	0.89 (0.55–1.46)	0.65		
Divorced or widowed	42/66 (63.64)	0.84 (0.49–1.44)	0.52		
Self-reported sexual orientation			0.31		
Homosexual	385/574 (67.07)	Ref.	Ref.		
Heterosexual	5/9 (55.56)	0.90 (0.45–1.81)	0.76		
Bisexual	127/206 (61.65)	1.14 (0.26–5.20)	0.86		
Not sure	20/33 (60.61)	1.04 (0.50–2.16)	0.93		
Ever had sex with women			0.00		
Yes	255/399 (63.91)	Ref.	Ref.	Ref.	Ref.
No	282/513 (54.97)	1.09 (0.84–1.43)	0.52	1.06 (0.75–1.51)	0.74
Age at the first homosexual act			0.00		
<18 years	87/220 (39.55)	Ref.	Ref.	Ref.	Ref.
≥18 years	447602 (74.25)	1.00 (0.74–1.35)	0.99	1.02 (0.71–1.46)	0.93
Ever had Genital wart			0.00		
No	60/85 (70.59)	Ref.	Ref.	Ref.	Ref.
Yes	477/737 (64.72)	0.71 (0.45–1.14)	0.12	0.75 (0.45–1.25)	0.27
Ever had other STDs			0.00		
No	83/118 (69.70)	Ref.	Ref.	Ref.	Ref.
Yes	453/704 (64.35)	0.84 (0.57–1.24)	0.38	0.85 (0.55–1.30)	0.45
Have current sexual partner			0.00		
No	224/345 (64.93)	Ref.	Ref.		
Yes	313/477 (65.62)	0.84 (0.57–1.24)	0.67	0.99 (0.72–1.36)	0.94
Frequency of homosexual behaviors in the past 6 months			0.00		
<once a week	401/615 (65.20)	Ref.	Ref.	Ref.	Ref.
≥once a week	105/207 (50.72)	1.15 (0.83–1.60)	0.41	1.01 (0.69–1.48)	0.96
Condom use during insertive anal sex in the past 6 months			0.00		
Always	359/548 (65.51)	Ref.	Ref.	Ref.	Ref.
Sometimes/Never	146/274 (53.28)	0.97 (0.72–1.31)	0.84	1.21 (0.87–1.70)	0.26
Number of homosexual partners in the past 6 months			0.00		
≤1	151/240 (62.92)	Ref.	Ref.	Ref.	Ref.
2–5	298/464 (64.22)	1.76 (1.12–2.77)	0.15	1.80 (0.99–3.24)	0.05
≥6	86/117 (73.50)	1.56 (1.02–2.37)	0.04	1.65 (1.00–2.73)	0.05
Number of homosexual partners ever had			0.00		
1–5	126/235 (53.62)	Ref.	Ref.	Ref.	Ref.
6–10	153/259 (59.07)	1.51 (0.98–2.35)	0.06	1.09 (0.62–1.92)	0.75
11–30	174/263 (66.16)	1.21 (0.79–1.87)	0.38	0.88 (0.52–1.48)	0.63
>30	84/132 (63.64)	0.90 (0.58–1.39)	0.02	0.71 (0.43–1.15)	0.16
Ever had multiple partner sex in the past year			0.00		
No	62/87 (71.26)	Ref.	Ref.	Ref.	Ref.
Yes	473/735 (64.35)	0.61 (0.38–0.99)	0.04	0.50 (0.29–0.87)	0.01
Ever had paid sex with man in the past year			0.00		
No	19/36 (52.78)	Ref.	Ref.	Ref.	Ref.
Yes	515/781 (65.94)	1.83 (0.97–3.43)	0.06	2.34 (1.16–4.74)	0.02
Ever found sexual partners in gay venues			0.00		
No	363/574 (63.24)	Ref.	Ref.	Ref.	Ref.
Yes	173/248 (69.76)	1.30 (0.96–1.75)	0.99	0.88 (0.36–2.20)	0.79

Abbreviation: CI, confidence intervals; OR, odds ratio; STD, sexual transmitted disease.

^*^Sum may not always add up to total because of missing data.

**Table 4:** Risk factors associated with prevalence of HIV-1 seropositivity.

Variables	Prevalence n/N* (%)	OR (95% CI)	*P-*value	Adjusted OR (95% CI)	*P-*value
Site			0.00		
Chengdu	40/281 (14.23)	Ref.			
Xi’an	41/256 (16.02)	1.92 (1.14–3.26)	0.02	1.93 (1.03–3.61)	0.04
Taiyuan	24/285 (8.42)	0.98 (0.62–1.56)	0.95	0.78 (0.45–1.35)	0.38
Age			0.00		
≤29 years	43/392 (10.97)	Ref.			
30–39 years	23/261 (8.81)	1.23 (0.75–2.04)	0.41	0.99 (0.45–2.22)	0.99
≥40 years	20/169 (11.83)	1.49 (0.86–2.60)	0.16	1.53 (0.75–3.13)	0.24
Education			0.00		
≤9 years	17/105 (16.19)	Ref.			
10–12 years	18/170 (10.59)	0.62 (0.33–1.16)	0.14	0.66 (0.32–1.35)	0.26
>12 years	51/547 (9.32)	1.31 (0.80–2.15)	0.29	1.21 (0.67–2.20)	0.53
Marriage status			0.00		
Unmarried	61/551 (11.07)	Ref.			
Married	19/205 (9.27)	1.24 (0.63–22,46)	0.54	0.97 (0.38–2.45)	0.95
Divorced or widowed	6/66 (9.09)	1.50 (0.69–3.24)	0.31	1.36 (0.55–3.34)	0.51
Self-reported sexual orientation			0.00		
Homosexual	65/574 (11.32)	Ref.			
Heterosexual	0/9 (0.00)	0.42 (0.10–1.77)	0.23	0.50 (0.11–2.27)	0.37
Bisexual	19/206 (9.22)	0.50 (0.04–6.23)	0.59	0.95 (0.06–14.15)	0.97
Not sure	2/33 (6.06)	0.51 (0.11–2.25)	0.37	0.62 (0.13–2.95)	0.55
Ever had sex with women			0.00		
Yes	39/87 (44.83)	Ref.			
No	47/735 (6.39)	1.17 (0.78–1.75)	0.45	1.44 (0.78–2.68)	0.24
Age at the first homosexual act			0.00		
<18 years	23/220 (10.45)	Ref.	Ref.	Ref.	Ref.
≥18 years	63/602 (10.47)	0.88 (0.56–1.36)	0.55	1.28 (0.74–2.22)	0.37
Ever had Genital wart			0.00		
No	17/85 (20.00)	Ref.	Ref.	Ref.	Ref.
Yes	69/737 (9.36)	0.43 (0.25–0.74)	0.00	0.49 (0.26–0.90)	0.02
Ever had other STDs			0.00		
No	20/118 (19.95)	Ref.	Ref.	Ref.	Ref.
Yes	66/704 (9.38)	1.01 (0.74–1.39)	0.00	0.70 (0.39–1.22)	0.20
Have current sexual partner			0.00		
No	28/345 (8.12)	Ref.	Ref.	Ref.	Ref.
Yes	58/477 (12.16)	1.43 (0.94–2.17)	0.10	1.49 (0.89–2.50)	0.13
Frequency of homosexual behaviors in the past 6 months			0.00		
<once a week	19/615 (3.09)	Ref.	Ref.	Ref.	Ref.
≥once a week	61/207 (29.47)	0.73 (0.45–1.17)	0.19	0.81 (0.46–1.42)	0.45
Condom use during insertive anal sex in the past 6 months			0.00		
Always	48/548 (8.76)	Ref.			
Sometimes/Never	33/274 (12.04)	1.83 (1.19–2.80)	0.01	1.55 (0.92–2.54)	0.08
Number of homosexual partners in the past 6 months			0.00		
≤1	22/240 (9.17)	Ref.	Ref.	Ref.	Ref.
2–5	48/464 (10.34)	1.35 (0.72–2.54)	0.34	0.85 (0.35–2.0)	0.70
≥6	16/117 (13.68)	1.23 (0.70–2.17)	0.48	0.83 (0.42–1.65)	0.59
Number of homosexual partners ever had			0.00		
1–5	20/235 (8.51)	Ref.	Ref.	Ref.	Ref.
6–10	19/259 (7.34)	1.53 (0.79–2.97)	0.21	1.51 (0.64–3.58)	0.35
11–30	34/263 (12.93)	1.48 (0.78–2.82)	0.24	1.96 (0.86–4.46)	0.11
>30	13/132 (9.85)	0.79 (0.43–1.42)	0.43	0.77 (0.39–1.55)	0.47
Ever found sexual partners in gay venues			0.00		
No	53/574 (9.23)	Ref.	Ref.	Ref.	Ref.
Yes	33/248 (13.31)	1.53 (1.01–2.31)	0.37	2.06 (0.87–4.87)	0.10
Ever had paid sex with man in the past year?			0.00		
No	19/36 (52.78)	Ref.	Ref.	Ref.	Ref.
Yes	67/781 (8.58)	1.80 (0.55–5.94)	0.33	2.01 (0.54–7.40)	0.30
Anal HPV infection (any type)			0.00		
Negative	19/286 (6.64)	Ref.	Ref.	Ref.	Ref.
Positive	86/536 (16.04)	2.73 (1.69–4.42)	0.00	3.03 (1.76–5.21)	0.00

Abbreviation: CI, confidence intervals; OR, odds ratio; STD, sexual transmitted disease.

^*^Sum may not always add up to total because of missing data.

**Table 5:** The association of anal HPV infection with HIV-1 seropositivity by oncogenic type and multiplicity of HPV types.

Anal infection of 37 HPV types	Prevalence of HIV-1 seropositivity n/N (%)	OR (95% CI)	Adjusted OR* (95% CI)
By oncogenic type			
Negative for any type	18/285(6.32)	Ref.	Ref.
Only Positive for low-risk type	13/118(11.02)	2.18 (1.30–3.66)	2.43 (1.42–4.16)
Only Positive for high-risk type	40/291(13.75)	2.81 (1.39–5.65)	2.91 (1.40–6.02)
Positive for both	30/124(24.19)	4.86 (2.64–8.96)	5.69 (3.00–10.81)
By multiplicity of HPV types			
0	18/285(6.32)	Ref.	Ref.
1	36/314(11.46)	2.38 (1.01–5.59)	2.64 (1.09–6.38)
2	18/120(15.00)	3.99 (1.84–8.63)	4.18 (1.88–9.31)
3	13/57(22.81)	5.43 (2.75–10.75)	5.59 (2.91–11.92)
≥4	19/46(41.30)	9.85 (4.66–20.84)	11.46 (5.25–25.00)
p for trend		<0.01	<0.01

Abbreviation: CI, confidence intervals; OR, odds ratio.

^*^Adjusted for age, ethnicity, education, age at the first homosexual act, frequency of homosexual behaviors in the past 6 months and ever found sexual partners in gay venues.
