# Supplementary material for: Anal HPV/HIV co-infection among Men Who Have Sex with Men: a cross-sectional survey from three cities in China
Source: Sci Rep. 2016 Feb 19;6:21368. doi: 10.1038/srep21368 (PMC4759533; doi:10.1038/srep21368)
Supplement: Supplementary Information [file srep21368-s1.pdf]

Anal HPV/HIV co-infection among Men Who Have Sex with Men: a cross-sectional survey from three cities in China

Xiangwei Li,[1#] Mufei Li,[1#] Yu Yang,[2] Xiang Zhong,[3] Boxuan Feng,[1] Henan Xin,[1] Zhen Li,[1] Qi Jin,[1*] Lei Gao[1*]

[1]MOH Key Laboratory of Systems Biology of Pathogens, Institute of Pathogen Biology, Chinese Academy of Medical Sciences & Peking Union Medical College, Beijing, China

[2]Department of Epidemiology and Biostatistics, School of Public Health, Peking University Health Science Centre, Beijing, China

[3]MSD China Holding Co., Ltd, Beijing, China

[#]These two authors contributed equally

Running title: HPV/HIV co-infection in MSM from China

[*]Correspondence:

Prof. Lei Gao and Prof. Qi Jin, Institute of Pathogen Biology, CAMS & PUMC. Dong Dan San Tiao 9.100730 Beijing, China. Phone: 86-10-67855003. Fax: 86-10-67855003. Email: gaolei@ipbcams.ac.cn (LG); jinqi@ipbcams.ac.cn (QJ)

Supplementary Table 1. Anal HPV type distribution by study site

HPV type	Total(N = 822)	By site			
		Chengdu (N=256)	Xi’an (N=281)	Taiyuan (N=285)	*p*-value
Any type	537(65.32)	135(52.73)	193(68.68)	209(73.33)	＜0.01
Single type	314(38.19)	87(33.98)	72(25.62)	155(54.39)	＜0.01
Multiple types	223(27.12)	48(18.75)	121(43.06)	54(18.95)	＜0.01
High Risk					
HPV 18	111(13.50)	38(14.84)	17(6.05)	56(19.65)	＜0.01
HPV 16	94(11.44)	22(8.59)	44(15.66)	28(9.82)	0.02
HPV 59	78(9.49)	23(8.98)	37(13.17)	18(6.32)	0.02
HPV 56	76(9.25)	10(3.91)	46(16.37)	20(7.02)	＜0.01
HPV 82	42(5.11)	2(0.78)	27(9.61)	13(4.56)	＜0.01
HPV 52	37(4.50)	9(3.52)	19(6.76)	9(3.16)	0.08
HPV 39	34(4.14)	3(1.17)	20(7.12)	11(3.86)	＜0.01
HPV 68	31(3.77)	9(3.52)	13(4.63)	9(3.16)	0.64
HPV 58	30(3.65)	5(1.95)	23(8.19)	2(0.70)	＜0.01
HPV 51	29(3.53)	5(1.95)	19(6.76)	5(1.75)	＜0.01
HPV 31	22(2.68)	7(2.73)	0	15(5.26)	＜0.01
HPV 66	21(2.55)	5(1.95)	13(4.63)	3(1.05)	0.02
HPV 45	17(2.07)	5(1.95)	12(4.27)	0	＜0.01
HPV 33	15(1.82)	2(0.78)	9(3.20)	4(1.40)	0.09
HPV 55	13(1.58)	5(7.95)	7(2.49)	1(0.35)	0.11
HPV 53	6(0.73)	1(0.39)	2(0.71)	3(1.05)	0.66
HPV 35	5(0.61)	0	5(1.78)	0	＜0.01
HPV 83	4(0.49)	2(0.78)	2(0.71)	0	0.34
HPV 26	1(0.12)	1(0.39)	0	0	/
HPV 57	1(0.12)	1(0.39)	0	0	/
HPV 34	0	0	0	0	/
HPV 69	0	0	0	0	/
HPV 71	0	0	0	0	/
Low Risk					
HPV 6	127(15.45)	27(10.55)	66(23.49)	34(11.93)	＜0.01
HPV 11	88(10.71)	26(10.16)	24(8.54)	38(13.33)	0.17
HPV 61	26(3.16)	8(3.13)	13(4.63)	5(1.75)	0.15
HPV 40	19(2.31)	5(1.95)	12(4.27)	2(0.70)	0.01
HPV 44	11(1.34)	3(1.17)	8(2.85)	0	0.01
HPV 84	3(0.36)	0	0	6(2.11)	＜0.01
HPV 42	6(0.73)	0	2(0.71)	1(0.35)	0.39
HPV 81	3(0.36)	1(0.39)	0	2(0.70)	0.38
HPV 54	2(0.24)	2(0.78)	0	0	/
HPV 43	0	0	0	0	/
HPV 67	0	0	0	0	/
HPV 70	0	0	0	0	/
HPV 72	0	0	0	0	/
HPV 73	0	0	0	0	/
